# Intraoperative Radiotherapy for Breast Cancer: The Lasting Effects of a Fleeting Treatment

**DOI:** 10.1155/2014/214325

**Published:** 2014-08-10

**Authors:** Harriet B. Eldredge-Hindy, Anne L. Rosenberg, Nicole L. Simone

**Affiliations:** ^1^Department of Radiation Oncology, Thomas Jefferson University Hospital, Sidney Kimmel Medical College of Thomas Jefferson University, 111 South 11th Street, Philadelphia, PA 19107, USA; ^2^Department of Surgery, Sidney Kimmel Medical College of Thomas Jefferson University, Philadelphia, PA 19107, USA

## Abstract

In well-selected patients who choose to pursue breast conservation therapy (BCT) for early-stage breast cancer, partial breast irradiation (PBI) delivered externally or intraoperatively, may be a viable alternative to conventional whole breast irradiation. Two large, contemporary randomized trials have demonstrated breast intraoperative radiotherapy (IORT) to be noninferior to whole breast external beam radiotherapy (EBRT) when assessing for ipsilateral breast tumor recurrence in select patients. Additionally, IORT and other PBI techniques are likely to be more widely adopted in the future because they improve patient convenience by offering an accelerated course of treatment. Coupled with these novel techniques for breast radiotherapy (RT) are distinct toxicity profiles and unique cosmetic alterations that differ from conventional breast EBRT and have the potential to impact disease surveillance and patient satisfaction. This paper will review the level-one evidence for treatment efficacy as well as important secondary endpoints like RT toxicity, breast cosmesis, quality of life, patient satisfaction, and surveillance mammography following BCT with IORT.

## 1. Introduction

Modern, randomized trials investigating breast conservation therapy (BCT) for early-stage breast cancer have shown that radiotherapy (RT) cannot be omitted in any subgroup of breast cancer patients without compromising local control [1–3]. However, it may be feasible to provide RT to only a portion of the breast in appropriately selected patients given that most local recurrences (LR) arise in the same region of the breast as the original tumor [4–12]. As such, there is considerable interest in developing techniques for partial breast irradiation (PBI) and identifying patient subgroups that may benefit from this approach. Proponents of PBI note several advantages of this therapy over conventional, whole breast external beam radiotherapy (EBRT) including both sparing of normal tissues and a shorter RT course that could improve patient convenience, compliance, and cost [7–12].

One type of PBI is intraoperative radiotherapy (IORT), which is typically delivered in a single fraction at the time of partial mastectomy (PM). Various techniques for breast IORT have been used for two decades; however, high-level clinical data have only recently emerged from randomized trials [7–9]. IORT is challenging the current standard in BCT in a selected patient population who are at low risk of LR and who are suitable for PBI [13]. With appropriate techniques, there may be a role for IORT. Emerging evidence demonstrates the noninferiority of this method and IORT may become more widely adopted with commensurate impact on the outcomes of BCT. The purpose of this review is to explore the manner in which IORT may impact important and lasting BCT endpoints like local control, RT toxicity, cosmetic outcome, quality of life (QOL), and surveillance mammography.

## 2. Randomized Evidence for Breast IORT

Data from observational series, small trials, and a systematic review have been reported and formed the basis for the two pivotal, international, randomized trials [7–22]. In fact, prior to the development of one of the randomized trials, the IORT technique was assessed in a phase II study in which IORT was delivered as a tumor bed boost and was followed by conventional, whole breast EBRT. Five-year estimates for ipsilateral breast tumor recurrence (IBTR) in 300 patients were notably lower (1.7%) than in contemporary clinical trials, prompting the hypothesis that an IORT boost might be superior to an EBRT boost [19]. These findings drove the development of the targeted intraoperative RT versus whole breast RT for breast cancer trial, also known as TARGIT-A trial [8, 9]. This was an international, phase III, randomized, noninferiority study in which a single fraction of targeted IORT was delivered using the Intrabeam system (Carl Zeiss, Oberkochen, Germany). This system delivers low-energy photons (maximum 50 kV) at the tip of a 3.2 mm diameter tube. Spherical applicators of various sizes cover the tube and are placed in the tumor bed. Radiation is subsequently administered in the operating room over 20–35 minutes. The surface of the applicator receives 20 Gy and absorbed dose rapidly attenuates to 5–7 Gy at 1 cm depth.

This trial enrolled 2,232 women who were at least 45 years of age with invasive ductal carcinoma and randomized them to receive either whole breast EBRT or targeted IORT following PM as well as sentinel node biopsy and/or axillary dissection [8, 9]. Women in the control arm were permitted to receive conventional EBRT either with or without a tumor bed boost. In the experimental arm, it was recognized that some patients would need whole breast EBRT in addition to targeted IORT because of prespecified, high-risk features found in the pathologic specimen following surgery, including invasive lobular carcinoma, extensive intraductal component, grade 3 tumor, and lymph node involvement. In the experimental arm, 86% of patients received targeted IORT alone while 14% received IORT followed by whole breast EBRT for these high-risk features. Furthermore, approximately one-third of patients were treated in parallel on a postpathology stratum in which women were enrolled and randomized after completion of PM and pathologic analysis of the surgical specimen. The postpathology women randomized to receive targeted IORT underwent a second surgical procedure to expose the lumpectomy cavity and deliver IORT at a median of 37 days following PM.

The primary endpoint of this trial was LR in the conserved breast with a prespecified noninferiority margin of an absolute difference of 2.5% in local control. Most women enrolled had tumors that were less than 2 cm (86%), are estrogen receptor positive (90%), and had negative lymph nodes (83%). Sixty-six percent received adjuvant endocrine therapy while 12% received chemotherapy. Outcomes were first reported in 2010 and the estimate of LR in the conserved breast at four years was 1.2% in the targeted IORT group and 0.95% in the EBRT group. This difference was not statistically significant (*P* = 0.41) and met the predefined noninferiority margin [8].

With a median follow-up of 2 years and 5 months, updated results for 3,451 women in the TARGIT-A trial demonstrated the 5-year LR to be 3.3% for targeted IORT versus 1.3% for whole breast EBRT (*P* = 0.042) [9]. While this difference was statistically significant, it achieved the predefined noninferiority endpoint. For reasons that are not understood, women who had received EBRT had a trend toward lower overall survival at five years (94.7% versus 96.1%, *P* = 0.099) and significantly higher rates of non-breast cancer death due to excess cardiovascular causes and other malignancies (3.5% versus 1.4%, *P* = 0.009).

A preplanned subgroup analysis of the pre- and postpathology strata was also performed on the TARGIT-A trial [9]. While targeted IORT given at the time of PM (prepathology stratum) provided similar local control to whole breast EBRT (LR 2.1% versus 1.1%; *P* = 0.310), delayed IORT (postpathology stratum) resulted in inferior rates of local control (LR 5.4% versus 1.7%; *P* = 0.069, absolute difference 3.7%). The authors speculated that the higher local control in the prepathology stratum was due to well-vascularized tissue suited for single-fraction therapy and an easily identified tumor bed at the time of surgery. Appropriately, the authors have cautioned against continued use of targeted IORT in the postpathology setting due to inferior rates of local control following BCT and, instead, only support the use of targeted IORT concurrent with PM [9]. Additionally, the authors have emphasized the risk-adapted approach of this trial in which EBRT was added to targeted IORT when high-risk features were present. Applying a risk-adapted approach may be the most practical way to implement IORT into clinical practice.

Despite the plethora of available data from the large TARGIT-A trial, it is unclear whether the results of this trial translate to other delivery systems for breast IORT including kilovoltage photon (e.g., Axxent and Intrabeam) and megavoltage electron devices (e.g., Mobetron and Novac 7) [16, 21–24].

The second notable randomized trial, the ELIOT trial, used an electron IORT system that was pioneered at the European Institute of Oncology in Milan [7]. With this approach, 21 Gy is administered in a single fraction using a portable linear accelerator with a robotic arm and electron energies ranging from 3 to 10 MeV. Flat and beveled applicators ranging from 3 to 12 cm in diameter are available, as are trolley-mounted and mobile lead devices for radiation shielding. Lead discs are placed behind dissected breast tissue to protect the intrathoracic organs during irradiation [15, 25]. While the administered dose is lower than the 60–66 Gy given with conventional EBRT, 21 Gy is biologically equivalent to 65 Gy when assuming an *α*/*β* ratio of 10 for breast tumor cells [7].

On the ELIOT trial, women with early breast cancer were randomized in a 1 : 1 ratio to receive either whole breast EBRT to 50 Gy followed by 10 Gy tumor bed boost or IORT to 21 Gy with electrons. Eligible women were between 48 and 75 years of age, had a maximum tumor diameter of 2.5 cm, and were candidates for BCT. This was an equivalence trial and the prespecified equivalence margin was a 7.5% rate of LR in the IORT arm, assuming a 3% rate of LR in the EBRT arm. In addition to PM, patients with a positive sentinel node biopsy underwent axillary dissection and, for patients with three or fewer positive lymph nodes, no additional irradiation was delivered. For patients with four or more positive lymph nodes, EBRT was postponed 8–12 weeks in the IORT group, although the dose and extent of this additional RT were not well described [7]. The primary study endpoint was IBTR, which was defined as a sum of LR at the lumpectomy site and second ipsilateral tumors occurring in any breast quadrant.

After a median follow-up of 5.8 years, 1,305 women were enrolled and the 5-year rate of IBTR in the IORT arm was 4.4%, including a LR rate of 2.5%. While the primary endpoint met the prespecified equivalence margin for IBTR, the occurrence of IBTR was significantly lower in the EBRT arm than had been originally estimated and the risk for IBRT was significantly different among the two study arms (4.4% versus 0.4%; *P* = 0.0001). Additionally, the 5-year occurrence of true LR in the index quadrant, new ipsilateral breast carcinomas, and regional lymph node relapse were significantly greater in the IORT group. In contrast, there was no significant difference in 5-year rates of contralateral breast cancers, distant metastasis, breast cancer-specific mortality, and overall survival between the two groups. Unlike in the TARGIT-A trial, there was no observed increase in death from nonbreast cancer causes in the EBRT group.

Due to the observed excess of IBTR in the IORT group of the ELIOT trial, authors reasoned that IORT could be incorporated into clinical practice using two approaches. First, IORT could be offered only to patients considered suitable based on all available preoperative diagnostic information like age, tumor size, receptor status, and tumor grade. The second scenario offers a risk-adapted approach similar to the TARGIT-A trial: full-dose IORT may be provided to well-selected patients at the time of PM and, after final pathologic analysis, additional whole breast EBRT may be provided to high-risk women. With this notion, IORT could be employed as a RT boost. However, authors concluded that IORT should be discussed with suitable patients desiring a personalized approach because it offers the advantage of a dramatically shortened treatment course. However, these advantages need to be weighed against the higher risk of IBTR following IORT [7].

Data from these IORT trials continue to challenge two areas of dogma in BCT: (1) that whole breast EBRT is necessary in all patients with invasive breast cancer and (2) that a higher, conventionally fractionated radiation dose is necessary for effective tumor control [8]. But dogma and reason can coexist and the results of these two contemporary IORT trials highlight the need for proper selection of patients prior to PBI [7–9, 26, 27]. For example, in the ELIOT trial, multivariate analysis of characteristics associated with local relapse revealed that tumor size greater than 2 cm, the presence of four or more positive lymph nodes, poorly differentiated tumors, and triple-negative subtype were independent risk factors for IBTR. Furthermore, when trial participants were grouped based on the presence of one or more of these risk factors, the risk of IBTR was 11.3% at five years compared to 1.5% in women with none of these features. These data are consistent with suitability criteria for accelerated PBI laid forth by the American Society for Radiation Oncology (ASTRO), as most patients with any number of these four characteristics would be classified as cautionary or unsuitable for accelerated PBI [28].

In a second series reported by investigators of the ELIOT trial, 1,822 patients treated with the ELIOT technique as the sole radiation modality after breast conserving surgery for invasive breast cancer were classified according to the ASTRO criteria for accelerated PBI into suitable, cautionary, and unsuitable groups [13]. The percentage of patients who met criteria for suitable, cautionary, and unsuitable groups was 16, 40, and 44, respectively. At five years, the rate of IBTR for suitable, cautionary, and unsuitable groups was 1.5%, 4.4%, and 8.8%, respectively (*P* = 0.0003), indicating that the ASTRO guidelines translate well to patients treated with the ELIOT technique as a monotherapy [13, 29]. The finding of a high LR for cautionary patients has been reproduced in other studies of electron IORT and when GEC-ESTRO guidelines for PBI are applied to the ELIOT population [30, 31]. However, it is challenging to apply the ASTRO guidelines for accelerated PBI towards selection of patients for IORT given that comprehensive pathologic information is not yet available while planning for or delivering IORT. One potential solution to this problem would be to obtain preoperative breast magnetic resonance imaging to identify women with multicentric disease. Additionally, core needle biopsy and intraoperative frozen section assessment could detect most pertinent pathologic information either before or at the time of surgery. With these modifications, the ASTRO recommendations could offer useful guidance to judge the appropriateness of PBI using the ELIOT technique [13].

Finally, while many patients with early-stage breast cancer are not suitable for breast IORT as a monotherapy, there remain discrete patient populations for which the technique may be particularly well suited. Keshtgar and colleagues have reported their experience of breast IORT with the TARGIT technique in 80 patients in whom EBRT was contraindicated for reasons such as prior breast RT, medical comorbidities, or autoimmune connective tissue disorders. After a median follow-up of 38 months, only two LR were observed, corresponding to an annual LR rate of 0.75% and demonstrating the appropriateness of IORT in this patient population [32]. Other series have investigated the use of breast IORT as a technique in the setting of reirradiation for IBTR [33, 34]. Finally, it has been shown that women over the age of 70 with small, estrogen positive tumors are at low risk for disease relapse and may benefit from an accelerated course of breast RT. The role of targeted IORT in women 70 years of age or older will need to be further evaluated in the upcoming TARGIT-E trial since elderly women comprised only a small subset of patients in the trial [35]. This will be a phase II, international trial to investigate the efficacy of IORT within elderly, low-risk patients undergoing BCT.

## 3. Breast IORT Leaves Its Mark: Toxicity and Mammographic Changes

The technique of breast IORT represents a marked deviation from conventional breast RT techniques in which small radiation doses are administered during each fraction. And while hypofractionated RT for breast cancer has gained popularity, fractional doses typically range from 2.66 to 5.7 Gy, which is dramatically lower dose than the 20-21 Gy administered during breast IORT [36–38]. Historically, large fraction sizes have been associated with increased late toxicity. As such, there is considerable interest in characterizing the late effects that follow the single, high doses used for breast IORT to ensure that this new modality has an acceptable toxicity profile and therapeutic window for routine clinical use.

Clinically significant toxicity was prospectively documented and assessed in the TARGIT-A trial (Table 1) [8]. Fortunately, the rate of major toxicity was low (range: 3.3–3.9%) and was similar among women treated with EBRT and targeted IORT. Other documented toxicities were similar between the arms of the trial and included low rates of hematoma requiring surgical evacuation, severe infection, and delayed wound healing. In the targeted IORT arm, the incidence of wound seromas requiring more than three aspirations was higher than following EBRT but, overall, this was an uncommon toxicity (2.1% versus 0.8%; *P* = 0.012). This event was compensated for by significantly lower severe (Radiation Therapy Oncology Group grade 3 or 4) radiation toxicity in the targeted IORT study arm (0.5% versus 2.1%; *P* = 0.002), indicating that the IORT is safe with no discernable increase in complication rate over conventional methods [8]. Similarly, skin toxicity was evaluated in a large subset of patients treated on the ELIOT trial but was not systematically documented as a trial endpoint for all patients (Table 1) [7]. Skin toxicities such as skin erythema, dryness, hyperpigmentation, and pruritus were milder and significantly less frequent among women in the IORT group. There were no documented cases of grade 3 or higher toxicity. Additionally, there were no identified differences in breast fibrosis, breast retraction, and breast pain among the study arms, although reported data are limited and the follow-up time was not extensive. Fat necrosis was detected on mammography in women treated on both trial arms but occurred twice as frequently in women who had received IORT (15% versus 7%; *P* = 0.04) (Table 2).

This toxicity of fat necrosis was described in early accounts of breast IORT [15]. For example, in a series of 590 patients treated with electron IORT, 2.5% of patients were noted to experience postoperative complications that could not be strictly classified as infection. This condition was characterized by a collection of brown fluid in the tumor bed with mild, overlying skin erythema and no signs of infection on fluid aspirate. Fat necrosis commonly occurred in the first month following surgery in older women with a higher proportion of fat tissue in the breast. In most cases, fat necrosis was self-limiting and, in rare cases, fat necrosis required surgical debridement or led to spontaneous dehiscence of the lumpectomy incision due to fluid pressure.

In addition to the data from prospective trials, data are also emerging which report additional toxicities that were not well evaluated in the TARGIT-A and ELIOT trials. For example, a recent study by Rampinelli and colleagues assessed pulmonary fibrosis detected with computed tomography following both breast EBRT and IORT using the ELIOT technique in 178 women [39]. Blinded reviewers scored fibrosis grade in a systematic manner. Not only was the rate of pulmonary fibrosis lower among women who received IORT (4% versus 46%; *P* < 0.0001), but the risk of any grade fibrosis was also 19 times higher following EBRT than IORT (OR 19.2, 95% CII 6.5–57.1). Furthermore, Leonardi and colleagues scored breast fibrosis following electron IORT and, after a median follow-up of six years, grade 2 and 3 fibrosis were 32% and 6%, respectively [40]. The authors reasoned that this rate of breast fibrosis is acceptably low, even after long-term follow-up.

Additionally, several other series document toxicity findings following breast IORT (Table 1). There is a considerable amount of heterogeneity in the reported prevalence of various toxicities in the literature which likely results from variations in IORT technique or dose, nonuniform systems for toxicity scoring, inconsistent follow-up length, and recall bias in retrospective series. In general, the most common reported toxicities following breast IORT are fat necrosis, seroma or fluid collections, and breast fibrosis or retraction. Skin toxicities like erythema, telangiectasias, and hyperpigmentation may occur following breast IORT but appear to be milder in severity in comparison with conventional whole breast EBRT [7, 41].

## 4. Surveillance

As new treatment methods like breast IORT evolve, there are also new aspects that need to be considered in the clinical and radiographic surveillance for disease recurrence. Specifically, it is important to understand whether IORT causes structural or radiographic changes within the breast that need to be accounted for on surveillance imaging. Potentially, posttreatment changes could mimic cancer recurrence and, indeed, breast IORT can cause parenchymal changes like fluid in wound cavities, calcifications, fat necrosis, and oil-like cysts within the breast (Table 2) [43–46, 48]. For example, in a subgroup analysis of patients treated on the TARGIT-A trial, mammographic changes following IORT were described. Overall, 258 mammograms were reviewed from 27 patients who received IORT and 21 patients who received whole breast EBRT. Posttreatment mammography demonstrated that fat necrosis occurred more frequently (56% versus 24%) and was larger (8.7 versus 1.6 cm^2^) following IORT than in controls. Additionally, scar calcifications occurred more frequently after IORT (63% versus 19%), which the authors speculated could contribute to diagnostic uncertainty [46].

Recently, Wasser et al. set out to determine whether these structural alterations following IORT significantly complicate follow-up mammographic evaluation [49]. In their study, 54 patients who received IORT were compared with a control group comprised of 48 women who underwent BCT with standard EBRT to the breast. Women who had undergone IORT were significantly more likely to have distinct mammographic changes at each time point evaluated during the first four years following therapy when compared to EBRT controls (52–62% versus 7–30%). These distinct changes included parenchymal scarring, hematoma, seroma, fat necrosis, and nonspecific dystrophic calcifications. Furthermore, blinded radiologists determined that these breast changes complicated evaluation of diagnostic mammography more commonly in the women who had received IORT, particularly two or more years following BCT. Despite these common breast changes and associated diagnostic uncertainty, the study did not show a discernable increase in the need for additional diagnostic studies like ultrasound and MRI.

Recently, changes that arise in the tumor bed after IORT were described using magnetic resonance (MR) mammography in a cohort of women who had received breast IORT as a monotherapy or in combination with EBRT [48]. In this study, several characteristic changes were noted following IORT including seroma-like lesions in 89% of patients and elements of fat necrosis in 81%. These findings were commonly associated with persistent, rim-like, contrast enhancement in the tumor bed that was highly prevalent in the early postoperative period, persisted during several years of follow-up, and diminished in size over several years following IORT. These MR findings were consistent with previous mammographic and ultrasound findings in which a partially organized wound cavity was present following IORT, accompanied by the development of fat necrosis over time. Importantly, the frequent changes in the tumor bed after IORT did not implicate uncertainty for diagnosis and, in most cases, could be easily differentiated from recurrent tumor due to subtle and smooth features of the enhancement in the tumor bed.

Conventionally fractionated breast RT delivered with EBRT is also known to cause radiographic changes in the months and years following BCT. Currently, only a few investigators have set out to compare mammographic and ultrasound changes in the breast following IORT with changes occurring following whole breast EBRT (Table 2) [7, 26, 43–46, 48]. For example, in one recent study, blinded radiologists reviewed breast radiography from 90 women at 6, 12, and 24 months after electron IORT or EBRT in order to describe the temporal response in the breast [43]. At each time point evaluated, women in the IORT group had markedly higher frequency and severity of breast distortion and edema. Breast distortion was commonly due to high rates of fat necrosis (44% versus 0% at 24 months) with or without associated calcifications, hypoechoic areas (22% versus 0% at 24 months), and fluid collections at the surgical site (36% versus 0% at 24 months). These collections commonly required fluid aspiration with resultant cytology revealing only necrotic tissue, a problem that has been documented in other studies [26, 43]. Additional studies have reported findings of prolonged seromas (19–42%), fat necrosis (11–56%), parenchymal scarring or fibrosis (43–95%), and calcifications (2–63%) following IORT with no discernable difference in radiographic changes following IORT with photons or electrons (Table 2) [7, 26, 43–46, 48].

Overall, current IORT follow-up data are limited to a handful of small, retrospective series and, therefore, there are still questions regarding the proper radiographic surveillance for women with early-stage breast cancer following IORT. Future, well-powered studies will be needed to fully characterize the severity and frequency of post-IORT radiographic changes and to determine whether they impede detection of locally recurrent tumors. Mature data from clinical trials should be forthcoming.

## 5. Cosmetic Outcomes

There are an increasing number of treatment options for early-stage breast cancer that have similar oncologic outcomes, so cosmetic outcomes have become increasingly important in the patient decision making process. Yet, the available data with which to counsel patients are limited to small series with short follow-up [11, 12, 14, 40, 47, 50]. Reported cosmetic outcomes are excellent or good in most women (range: 77–100%) following breast IORT with commensurate levels of patient satisfaction (Table 3). When reported, adverse cosmetic effects include telangiectasias and fibrotic changes that can alter breast size, shape, and symmetry.

Investigators from Montpellier reported detailed cosmetic outcomes following IORT from a phase II trial [11]. In this trial, 42 patients 65 years or older with stage I breast cancer and negative surgical margins were treated with 21 Gy using electron IORT and no further RT. Posttreatment mammograms revealed fat necrosis in 71% of patients with 40% of those noted to have an underlying palpable mass which, unfortunately, complicated evaluation of mammograms. Physicians used clinical exams, systemic photography, and patient questionnaires to assess cosmetic outcome for 60 months. Although there were prevalent mammographic changes, this did not translate into uniformly poor cosmesis. Patients reported that IORT altered breast size and shape in 29% and 46% of patients, respectively. Nipple position and shape was chronically altered in only seven percent of patients and no chronic hyperpigmentation or telangiectasias were noted. Despite some documented cosmetic changes, most chronic findings were mild and moderate in severity with overall cosmesis being excellent or good in 85% of patients at five years.

In another study with well-documented aesthetic outcomes, women were objectively analyzed to determine whether targeted IORT leads to damaging fibrosis and poor cosmesis [50]. Frontal digital photographs were taken in 342 women at baseline and annually following treatment with either conventional EBRT or single-fraction IORT using the TARGIT method, which were then analyzed by the Breast Cancer Conservative Treatment Cosmetic Results software (BCCT.core). This software assesses breast symmetry, color, and scar. Following generation of a composite score, women are deemed to have excellent, good, fair, or poor cosmesis. Women who were treated with targeted IORT were found to have a 2.48-fold statistically significant increase in the odds of having a good or excellent outcome at two years compared to those treated with conventional EBRT. This trend persisted after adjusting for patient age, tumor size, and other clinical features. Also, breast asymmetry was noted to be greater in women who received EBRT throughout the five years of follow-up, likely due to lower scores for skin color and scar appearance. Overall, the main determinants of aesthetic outcome were differences in skin erythema and breast asymmetry between the treatment arms [51]. The excellent cosmesis observed in the IORT arm may have been due to the smaller volume of breast tissue irradiated during IORT, which may cause less fibrosis and asymmetry. Additionally, when IORT is delivered from within the breast, the negative effects of RT to the skin and underlying breast tissue appear to be minimized [50].

## 6. Is There Quality with Convenience? Quality of Life following IORT

In theory, IORT offers many potential advantages over conventional breast EBRT that may improve QOL in women with breast cancer [27]. For example, widespread implementation of IORT could dramatically shorten the RT treatment course from 5-6 weeks in conventional breast EBRT to a single intraoperative treatment in well-selected women. A shorter treatment course could translate into a shorter and milder acute toxicity phase and is undoubtedly more convenient. IORT is also known to be significantly less expensive than whole breast EBRT and PBI with brachytherapy, which may lead to less out-of-pocket cost incurred by the patient [14]. Despite the numerous potential advantages of PBI with IORT, there is a paucity of data surrounding health-related QOL outcomes.

Given that IORT may be used either as a RT boost in conjunction with EBRT or as a monotherapy following breast conservation surgery, it is possible that QOL may be impacted to varying degrees. Fortunately, Lemanski and colleagues have explored QOL parameters following both treatment paradigms [11, 12, 51]. In an early study, investigators reported QOL in 50 women following 9–20 Gy IORT boost and 50 Gy breast EBRT using the European Organization for Research and Treatment of Cancer QOL questionnaires-C30 (QLQ-C30), which evaluates five functional scales (physical, role, cognitive, emotional, and social), three symptom scales (fatigue, nausea, and pain), and a global health scale, and BR23 (QLQ-BR23) which evaluates four functional scales (body image, sexual function, sexual enjoyment, and future perspective) and four symptom scales (systemic side effects, breast symptoms, arm symptoms, and upset by hair loss) [51]. After a median follow-up of nine years, function and symptom scores were excellent. Results of QLQ-23 symptom scales revealed that breast and arm symptoms were the most commonly reported; however, outcome scores were low and ranged from 4 to 11 on a 100-point scale, indicating that symptoms were mild in severity. Patients who experienced subcutaneous fibrosis in the breast were more likely to report painful symptoms.

In separately published series, Lemanski and colleagues reported on QOL outcomes among women who had received 21 Gy IORT with electrons as a monotherapy following PM on a phase II trial in which QOL was a preplanned secondary endpoint [11, 12]. Again, QLQ-C30 and QLQ-BR23 questionnaires were used to assess outcomes, and resultant scores indicated excellent QOL (functional score range: 81–99) with few residual symptoms (symptom score range: 0–11) in the years that followed IORT. Scores for body image were high and there was no discernable change from baseline in patients' functional status and activities of daily living [12].

Another study by Welzel and colleagues directly compared QOL outcomes among women who received adjuvant breast RT using various techniques [52, 53]. The most recent series reported on a cohort of 230 women, including 25 who received IORT alone, 106 who received IORT in conjunction with WBRT, and 99 who received EBRT [53]. QOL data was collected using the QLQ-C30 and QLQ-BR23 questionnaires and, among scored outcomes for general pain, breast, and arm symptoms, scores were significantly more favorable for IORT than EBRT. QOL outcomes following IORT alone were also better than in women who received both IORT boost and EBRT. When present, these symptoms were characterized by pain in the treated area of the affected breast, difficulty in raising or moving the ipsilateral arm, and pain in the ipsilateral shoulder. Similar to these findings, a recent institutional series by Andersenet al. included 238 patients who were enrolled and treated on the TARGIT-A trial. Persistent pain after breast cancer treatment was found to be lower in the IORT group (25%) when compared to the breast EBRT group (34%), with a trend toward significance (*P* = 0.11) [54].

In addition to discomfort symptoms in the arm and breast, the Welzel series investigated additional comprehensive parameters. For example, women treated with IORT alone were found to have fewer restrictions in daily activities and better role functioning following RT than those women treated with EBRT. In contrast, there were no notable differences when additional endpoints were assessed among the treatment subgroups, including measures of anxiety and depression, cancer therapy-related fatigue, self-esteem, and body image [52, 53].

Currently, important radiation-related QOL parameters following IORT appear to be acceptable. While limited, the current literature supports that symptoms following IORT alone are mild and generally limited to localized breast or shoulder pain. Functional status appears to be minimally impacted by breast IORT and QOL outcomes may be more favorable following IORT alone when compared to techniques that incorporate breast EBRT.

## 7. Conclusions

There is a growing body of evidence supporting PBI for carefully chosen women with early breast cancer. Two contemporary clinical trials have established IORT as noninferior to conventional, whole breast EBRT methods. However, the convenience of this one-time treatment administered during breast conservation surgery should be weighed against the slightly higher risk of IBTR that has been noted in randomized trials of IORT. Clinical trial data with long-term follow-up are needed to determine whether the noninferior local control is a durable finding. It has been established that breast IORT has a distinct toxicity profile marked by liponecrosis, seroma formation, calcifications, and fibrotic changes. While frequently asymptomatic, the diagnostic implications of these parenchymal changes in the context of breast cancer surveillance are not fully understood. Yet, early reports of cosmetic outcomes and QOL following breast IORT are excellent and compare favorably to outcomes with conventional EBRT. Patient satisfaction, toxicity, and health-related QOL are increasingly important endpoints in breast clinical trials and should be continually investigated as IORT data mature. These data enable clinicians to understand the manner in which patients perceive their cancer treatments and disease outcomes.

## Figures and Tables

**Table 1:** Reported clinical toxicities following breast intraoperative radiotherapy in selected large series.

Series	Patients	Year	IORT^∗^ dose	IORT technique	Outcome time point (months)	Fat necrosis (%)	Seroma (%)	Fibrosis (%)	Skin (%)	Edema (%)	Infection (%)
TARGIT-A trial [8]	1113	2010	20 Gy	kV photons	NR	NR	2.1^†^	NR	2.8^‡^	NR	1.8
Veronesi et al. [29]	1822	2010	21 Gy	Electrons	36	4.2	12.9	1.9	NR	1.3	1.3
ELIOT trial [7]	651	2013	21 Gy	Electrons	70	15.0	NR	NR	1.5	NR	NR
Sperk et al. [41]	305	2012	20 Gy ± EBRT	kV photons	36	NR	NR	17.0	5.8	1.9	NR
Grobmyer et al. [14]	80	2013	20 Gy	Photons	13	NR	32.5	1.0	4.7	NR	5.0
Sacchini et al. [17]	52	2008	18–20 Gy	Brachytherapy	31	NR	50.0	NR	56.0	NR	8.0
Mussari et al. [42]	47	2006	20–24 Gy	Electrons	36	2.0	34.0	32.0	4.0	2.0	NR

^∗^IORT: intraoperative radiotherapy; Gy: gray; kV: kilovoltage; NR: not reported; EBRT: external beam radiotherapy.

^†^This study reported recurrent seroma.

^‡^This study reported only major skin toxicity.

**Table 2:** Selected series comparing the radiographic changes that follow breast intraoperative radiotherapy and whole breast external beam radiotherapy.

Series	Patients	Year	RT^∗^ method	IORT technique	Imaging	Outcome time point (months)	Fat necrosis (%)	Seroma (%)	Fibrosis (%)	Calcs. (%)	Edema (%)
Veronesi et al. [7]	651	2013	IORT alone	Electrons	MMG and US	70	15	NR	NR	NR	NR
			EBRT				7	NR	NR	NR	NR
Della Sala et al. [43]	90	2006	IORT alone	Electrons	MMG and US	6, 12, 24	11, 42, 44	31, 42, 36	NR	2, 18, 20	50, 7, 0
			EBRT			6, 12, 24	7, 4, 0	11, 4, 0	NR	2, 2, 7	25, 2, 0
Carvalho et al. [44]	60	2011	IORT alone	Electrons	MMG	12, 24	20, 33	NR	43, 47	NR, 60	37, 27
			EBRT			12, 24	7, 13	NR	37, 23	NR, 47	57, 40
Wasser et al. [45]	54	2007	IORT + EBRT	kV photons	MMG and US	24	52	33	93	22	89
			EBRT			24	15	4	93	7	96
Sautter-Bihl et al. [26]	53	2010	IORT + EBRT	Electrons	MMG	NR	50	NR	95	NR	75
			EBRT			NR	18	NR	88	NR	91
Engel et al. [46]	48	2013	IORT ± EBRT	kV photons	MMG	52	56	19	85	63	NR
			EBRT			52	24	0	95	19	NR

^∗^RT: radiotherapy; IORT: intraoperative radiotherapy; calcs.: calcifications; EBRT: external beam radiotherapy; MMG: mammogram; US: ultrasound; NR: not reported; kV: kilovoltage.

**Table 3:** Selected series reporting cosmetic outcome following breast intraoperative radiotherapy.

Series	Patients	Year	IORT^∗^ dose	IORT technique	Outcome time point (months)	Scoring method	Excellent cosmetic outcome (%)	Good cosmetic outcome (%)	Fair or poor cosmetic outcome (%)	Patient satisfaction (%)
Grobmyer et al. [14]	78	2013	20 Gy	kV photons	18	HCS	81	19	0	NR
Lemanski et al. [11]	42	2013	21 Gy	Electrons	60	Physician assessment, patient questionnaire	21	64	11	98
Leonardi et al. [40]	119	2012	21 Gy	Electrons	71	LENT-SOMA	77	NR	23	90
Kimple et al. [47]	71	2011	15 Gy	Electrons	12	Patient reported	43	33	24	87

^∗^IORT: intraoperative radiotherapy; Gy: gray; kV: kilovoltage; HCS: Harvard Cosmetic Scale; NR: not reported.

## Abbreviations

ASTRO: American Society for Radiation Oncology
BCT: Breast conservation therapy
EBRT: External beam radiotherapy
ELIOT: Intraoperative radiotherapy versus external radiotherapy for early breast cancer trial
GEC-ESTRO: Groupe Européen de Curiethérapie and the European Society for Radiotherapy and Oncology
Gy: Gray
IBTR: Ipsilateral breast tumor recurrence
IORT: Intraoperative radiotherapy
kV: Kilovoltage
LR: Local recurrences
PB: Partial breast irradiation
PM: Partial mastectomy
QOL: Quality of life
RT: Radiotherapy
TARGIT: Targeted intraoperative radiotherapy versus whole breast radiotherapy for breast cancer trial.
